# P-2315. Clinical outcome of rifabutin-based treatment for pulmonary tuberculosis in solid organ transplant recipients

**DOI:** 10.1093/ofid/ofae631.2467

**Published:** 2025-01-29

**Authors:** Jinyoung Yang, Jae-Hoon Ko, Sun Young Cho, Cheol-In Kang, Doo Ryeon Chung, Kyong Ran Peck, Jinsoo Rhu, Jong Man Kim, Gyu-Seong Choi, Kyo Won Lee, Jae Berm Park, Woo Seong Huh, Darae Kim, Jin-Oh Choi, Kyungmin Huh

**Affiliations:** Samsung Medical Center, Seoul, Seoul-t'ukpyolsi, Republic of Korea; Samsung Medical Center, Seoul, Seoul-t'ukpyolsi, Republic of Korea; Samsung Medical Center, Seoul, Korea, Seoul, Seoul-t'ukpyolsi, Republic of Korea; Samsung Medical Center, Seoul, Seoul-t'ukpyolsi, Republic of Korea; samsung medical center, Seoul, Seoul-t'ukpyolsi, Republic of Korea; Samsung Medical Center, Seoul, Seoul-t'ukpyolsi, Republic of Korea; Samsung Medical Center, Sungkyunkwan University School of Medicine, Seoul, Seoul-t'ukpyolsi, Republic of Korea; Samsung Medical Center, Sungkyunkwan University School of Medicine, Seoul, Seoul-t'ukpyolsi, Republic of Korea; Samsung Medical Center, Sungkyunkwan University School of Medicine, Seoul, Seoul-t'ukpyolsi, Republic of Korea; Samsung Medical Center, Sungkyunkwan University School of Medicine, Seoul, Seoul-t'ukpyolsi, Republic of Korea; Samsung Medical Center, Sungkyunkwan University School of Medicine, Seoul, Seoul-t'ukpyolsi, Republic of Korea; Samsung Medical Center, Sungkyunkwan University School of Medicine, Seoul, Seoul-t'ukpyolsi, Republic of Korea; Samsung Medical Center, Sungkyunkwan University School of Medicine, Seoul, Seoul-t'ukpyolsi, Republic of Korea; Samsung Medical Center, Sungkyunkwan University School of Medicine, Seoul, Seoul-t'ukpyolsi, Republic of Korea; Samsung Medical Center, Sungkyunkwan University School of Medicine, Seoul, Seoul-t'ukpyolsi, Republic of Korea

## Abstract

**Background:**

Rifabutin (Rfb) is commonly administered instead of rifampicin for the treatment of tuberculosis (TB) in SOTR to avoid substantial drug interaction of rifampin. However, evidence of its efficacy is scarce.

**Figure:**
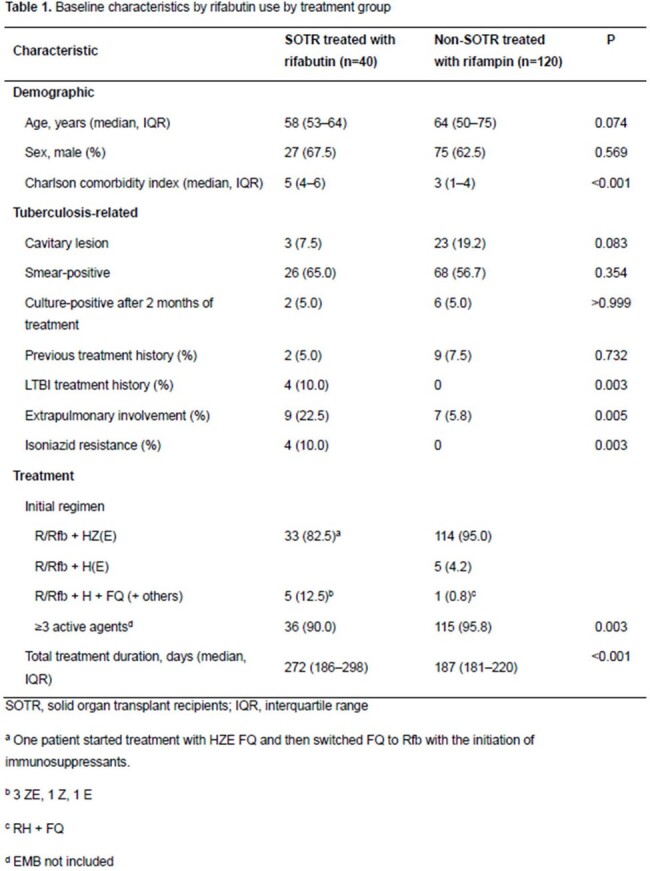

**Methods:**

We conducted a retrospective, case-control study at Samsung Medical Center. The SOTR group included SOTR aged ≥ 18 years who were diagnosed with culture-positive pulmonary TB and treated with anti-TB medications that included both isoniazid (INH) and Rfb for > 80% of the total treatment duration. Patients who discontinued immunosuppressants before TB diagnosis and those with rifampin-resistant TB were excluded. The SOTR group was matched with a 1:3 ratio to the non-SOTRs who were treated with rifampin-based regimens. The primary outcome was survival treatment completion without early relapse. Logistic regression with and without overlap weighting was used to evaluate outcome measures.

**Figure:**
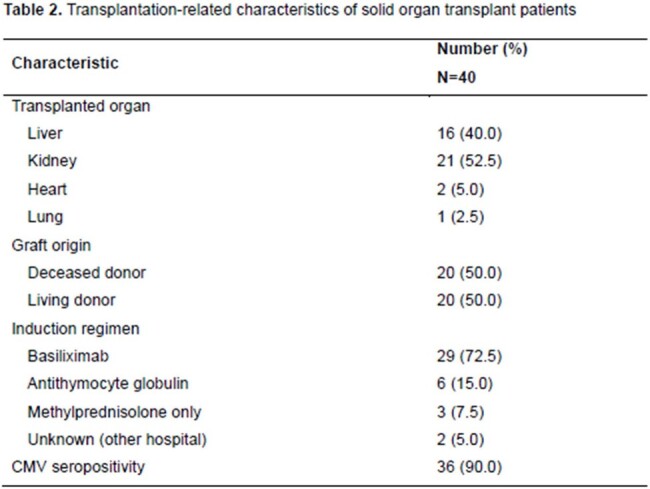

**Results:**

A total of 40 SOTRs and 120 non-SOTRs were enrolled. There was no difference between the two groups in the presence of cavitary lesions, smear-positivity, or culture-positivity at two months. However, extrapulmonary TB and INH resistance were significantly more frequent in the SOTR group. Most SOTRs had kidney transplants, followed by liver transplants. The proportions of deceased donors and living donors were 50% each. The primary outcome was achieved in 90% vs. 96.7% in SOTRs and controls, respectively (P=0.108). However, the completion of treatment was more frequent in the non-SOTR group (92.5% vs. 100%, P=0.015). There was no difference in recurrence or all-cause mortality within 1 year of completion of treatment, and there was no TB-attributable mortality in both groups. There was no significant difference in primary outcome after overlap weighting (adjusted odds ratio, 0.31; 95% confidence interval, 0.07–1.30). Grade 3 or higher acute kidney injury or hepatotoxicity was significantly more common in the SOTR group. In the SOTR group, allograft rejection requiring anti-rejection therapy within 6 months of TB diagnosis occurred in 10% and allograft failure in 12.5%.

**Figure:**
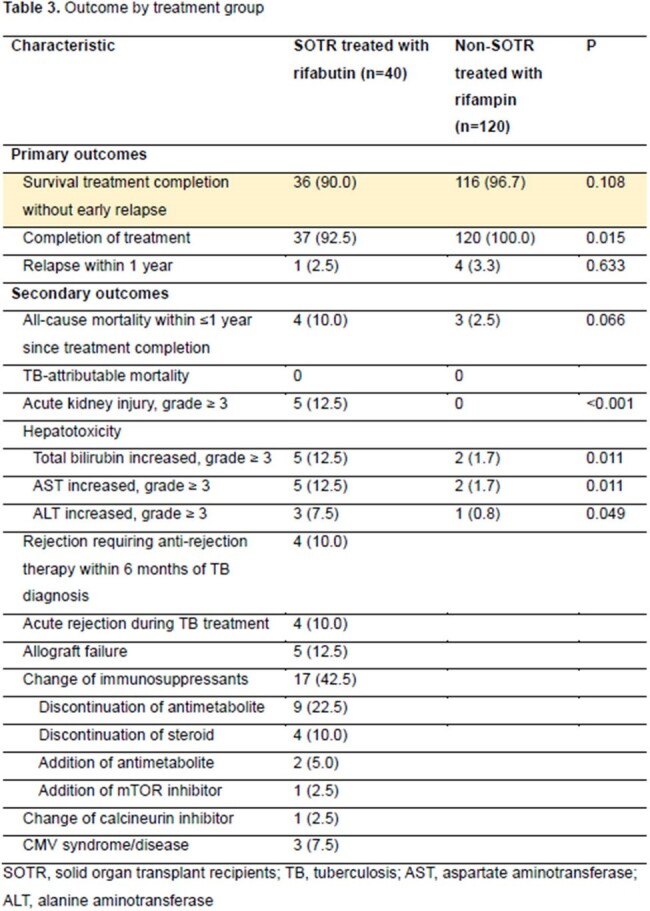

**Conclusion:**

Treating TB using Rfb in SOTR showed a similar treatment outcome compared to rifampin-based treatment in non-SOTR, suggesting its efficacy in this population.

**Figure:**
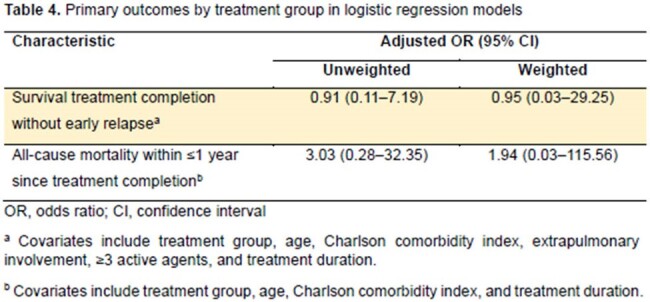

**Disclosures:**

Kyungmin Huh, M.D., Ph.D., bioMérieux: Grant/Research Support|GSK Korea: Advisor/Consultant|Pfizer Korea: Honoraria|Takeda Korea: Advisor/Consultant

